# Extracellular Protease Inhibition Alters the Phenotype of Chondrogenically Differentiating Human Mesenchymal Stem Cells (MSCs) in 3D Collagen Microspheres

**DOI:** 10.1371/journal.pone.0146928

**Published:** 2016-01-13

**Authors:** Sejin Han, Yuk Yin Li, Barbara Pui Chan

**Affiliations:** Tissue Engineering Laboratory, Department of Mechanical Engineering, The University of Hong Kong, Pokfulam Road, Hong Kong Special Administrative Region, China; University of Maryland School of Medicine, UNITED STATES

## Abstract

Matrix remodeling of cells is highly regulated by proteases and their inhibitors. Nevertheless, how would the chondrogenesis of mesenchymal stem cells (MSCs) be affected, when the balance of the matrix remodeling is disturbed by inhibiting matrix proteases, is incompletely known. Using a previously developed collagen microencapsulation platform, we investigated whether exposing chondrogenically differentiating MSCs to intracellular and extracellular protease inhibitors will affect the extracellular matrix remodeling and hence the outcomes of chondrogenesis. Results showed that inhibition of matrix proteases particularly the extracellular ones favors the phenotype of fibrocartilage rather than hyaline cartilage in chondrogenically differentiating hMSCs by upregulating type I collagen protein deposition and type II collagen gene expression without significantly altering the hypertrophic markers at gene level. This study suggests the potential of manipulating extracellular proteases to alter the outcomes of hMSC chondrogenesis, contributing to future development of differentiation protocols for fibrocartilage tissues for intervertebral disc and meniscus tissue engineering.

## Introduction

The process of chondrogenesis occurs in stages beginning with mesenchymal cell condensation followed by chondrocyte differentiation and maturation [1]. This process is characterized by a series of differentiation stages with extracellular matrix (ECM) remodeling. In particular, the stage-specific changes of pre-cartilaginous ECM containing fibronectin and type I collagen to cartilaginous ECM containing type II collagen and aggrecan as chondrocytic cells differentiate [2], and then to a matrix rich in type X collagen during terminal differentiation of chondrocytes [1].

Matrix remodeling involving degradation of the old ECM and deposition of the new ECM is important for tissue dynamic processes such as development, homeostasis and wound healing. The process is highly regulated by proteases and their inhibitors [3]. Transgenic mouse model suggested that the loss of membrane-type 1 MMP proteolytic activity in unmineralized cartilage affects skeletal development by impairing collagen remodeling [4,5]. Moreover, matrix metalloprotease-2 (MMP-2) regulated mesenchymal cell condensation by modulating the fibronectin matrix [6]. These studies suggest that processes involving matrix remodeling such as cell differentiation may be able to be manipulated by interfering with the balance of matrix degradation.

We previously developed a collagen microencapsulation platform where mesenchymal stem cells (MSCs) are entrapped in a reconstituted nanofibrous meshwork of type I collagen [7]. This type I collagen meshwork provides a good pre-cartilageous matrix template for the MSC to remodel during chondrogensis, via simultaneous deposition of new cartilageous matrix rich in type II collagen and proteoglycans, and degradation of the old type I collagen matrix [8,9]. This provides a good in vitro model to study the chondrogenesis process.

In the current study, we hypothesize that treatment of intracellular and extracellular protease inhibitors will affect the matrix remodeling of MSC during chondrogenesis. The significance of this study is to manipulate the outcome of MSC chondrogenesis by modulating the matrix degradation, so as to contribute to cartilage tissue engineering.

## Materials and methods

### Culture of human mesenchymal stem cells (hMSCs)

Human MSCs from bone marrow [10] were kindly provided by Dr. G.C.F. Chan, Department of Paediatrics and Adolescent Medicine, The University of Hong Kong and cultured as monolayers as previously described [10], according to a protocol approved by the Combined Clinical Ethics Committee of the University of Hong Kong and Hong Kong West Cluster Hospitals of Hospital Authority. In brief, hMSCs were cultured in growth medium (Dulbecco’s modified Eagle’s medium-low glucose (DMEM-LG), 10% fetal bovine serum (FBS), 100 U/ml penicillin, 100 mg/ml streptomycin and 2 mM L-glutamine) at 37°C in a humidified atmosphere with 5% CO_2_. The growth medium was replaced every 3–4 days. At around 80% confluence, hMSCs were isolated by trypsinization with trypsin-EDTA (0.05%) briefly before re-suspending in full medium for subsequent experiments. Cells at P6 were used for subsequent experiments.

### Encapsulation of hMSCs into type I collagen scaffold

Human mesenchymal stem cells (hMSCs) at passage 6 were used for the subsequent experiments. When hMSCs were ~80% confluent, cells were prepared for encapsulation into collagen scaffold as described previously [7]. Briefly, cells were trysinized with 0.05% trypsin-EDTA. Cell suspension were mixed with neutralized rat tail type I collagen solution obtained from commercial source (BD Biosciences, Bedford, MA) [7] in an ice-bath with two parameters of cell seeding density (4 x 10^6^ cells/ml) and collagen concentration (1 mg/ml). Droplets of the mixtures of various volumes (100 μl) were pipetted into petri-dishes with UV-irradiated parafilm covering the bottom of each dish, to prevent adhesion of the constructs to the substratum. The collagen—hMSC mixtures were gelated when incubated at 37°C in a humidified atmosphere with 5% CO_2_ for 45 min. The gelated droplets were then free-floated in growth medium to allow contraction for 3 days. At day 3 after encapsulating hMSCs into collagen microspheres, microspheres were moved into 24-well dishes covered with parafilm.

### Chondrogenic differentiation of hMSC in collagen microspheres

Chondrogenic differentiation of hMSC-collagen microspheres was induced in the absence of serum using the well-established culture condition [11] by replacing culture medium with chondrogenic differentiation induction medium (CM) at the third day after encapsulation. CM was defined as Dulbecco’s modified Eagle’s medium-high glucose (DMEM-HG), supplemented with 10 ng/ml recombinant human TGF-β3 (Merck, Darmstadt, Germany), 100 nM dexamethasone (Sigma, St. Louis, MO, USA), 0.1 mM L-ascorbic acid 2-phosphate (Fluka, St. Louis, MO, USA), 6 mg/ml insulin (Merck), 6 mg/ml transferrin (Sigma), 1 mM sodium pyruvate (Gibco, Grand Island, NY, USA), 0.35 mM L-proline (Merck), 100 U/ml penicillin, 100 μg/ml streptomycin and 2 mM L-glutamine) and 1.25 mg/ml bovine serum albumin (BSA) (Sigma). CM was freshly prepared and regularly changed every 2 days for 4 weeks.

### Intracellular and extracellular protease inhibition

At the day 0 of chondrogenic differentiation induction, hMSC-collagen microspheres were divided in inhibitor treatment groups. Microspheres were maintained in CM for 28 days; each group treated with protease inhibitors at an optimal concentration previously determined: E-64D (Aloxistatin, Cayman chemical, 20mM) and/or GM6001 (Ilomastat, Millipore, 25mM). Inhibitor stock solutions were prepared with DMSO at the concentration of 1mg/ml. One control group was treated with DMSO alone to check the effect of DMSO. The medium with inhibitors was freshly prepared and changed three times a week.

### Histological, immunohistochemical (IHC), and immunofluorescence (IF) analyses

Samples were fixed at desired time points with 4% PBS-buffered paraformaldehyde for 10 min at room temperature and cut into frozen sections (12 μm) and paraffin sections (5 μm). The sections were stained with hematoxylin and eosin (H&E) for histological analyses, or stained with Alcian blue to visualize any glycosaminoglycan (GAG) deposit. The sections were permeabilized with 0.02% TritonX-100 for 10 min and depending on antigen, antigens were retrieved by pepsin or hyaluronidase and pronase, then endogenous peroxidase activity was blocked with H_2_O_2_. Sections were blocked with 2% normal horse serum for non-specific binding and then incubated with primary antibody against type I collagen, type II collagen, type X collagen, Sox9, and aggrecan overnight at 4°C. Secondary antibody and avidin—biotin—peroxidase complex (Vector Laboratories) with DAB substrate system (Dako) were used according to the suppliers’ instructions. The sections were counterstained in hematoxylin. Negative control was performed without primary antibodies under identical conditions. Table 1 shows the antibody dilution ratio for IHC.

**Table 1:** The antibody dilution ratio for IHC.

Antibody	Primary	Secondary
**Type I Collagen (Sigma Aldrich, c2456)**	10000	2000
**Type II collagen (Calbiochem)**	1000	200
**Type X collagen(ab58632, ab49945)**	500	200
**Aggrecan(ab3778)**	1000/500	200/200
**SOX9 (sc20095)**	500	200
**SOX9 (ab76997)**	1000	200

For immunofluorescent analysis, after overnight incubation with primary antibodies at 4°C, sections were further incubated with Alexa fluor-488, 546, 647 conjugated secondary antibody in dark for 1 h at room temperature. Sections were mounted with anti-fading fluorescent mounting medium with DAPI (Electron Microscopy Sciences, Hatfield, PA, USA). Table 2 shows the antibody dilution ratio for IF.

**Table 2:** The antibody dilution ratio for IF.

Antibody	Primary	Secondary
**Type I Collagen (Sigma Aldrich, c2456)**	400	200
**Type II collagen (Calbiochem)**	100	400
**Type X collagen (ab58632, ab49945)**	100	200
**Aggrecan(ab3778)**	100	200
**Aggrecan(sc25674)**	100	400
**SOX9 (sc20095)**	50	400
**SOX9 (ab76997)**	100	400
**MMP2(sc8835)**	100	200

### Measurement of GAG content

The microspheres were digested overnight with 300 μg/ml papain in 50 mM phosphate buffer (pH 6.5), containing 5 mM cysteine and 5 mM EDTA at 60°C. The amount of GAG accumulation in the microspheres was determined by the 1, 9-dimethylmethylene blue (DMMB) method [12]. GAG concentration was calculated by calibrating against a standard curve obtained with shark chondroitin sulfate (Sigma). To assess the biosynthetic activity of the cells, results of GAG quantification were normalized to the total protein represented by HYP or DNA content, which was quantified by a fluorometric assay with Hoechst 33258 [13]. The DNA content was determined against a standard curve of calf thymus DNA (Sigma). Both the GAG and the DNA assays were run in triplicate for each group. Data were expressed as mean ± standard deviation.

### Hydroxproline (HYP) assay

To determine the content of HYP, which is the marker for collagen, 2 to 3 microspheres were digested in a 100 μl digestion solution (pH 6.5) consisting of 50 mM phosphate buffer (Sigma), 5 mM EDTA (Sigma), 5 mM L-cysteine (Sigma), and 300 μg/ml papain (Sigma) at 60°C overnight, as described previously [9]. Then a digested sample was hydrolyzed with 6 N HCl at 110°C for 18 h in a hydrolysis tube (Pierre) after being flushed with nitrogen gas for 30 s and was neutralized by 6 N NaOH (pH 6~7). Neutralized samples were incubated with 50 μl of 0.05 M chloramine T solution (Sigma) for 20 min and oxidized by 50 μl of 3.15M perchloric acid (Sigma) for 5 min and finally mixed with 50 μl p-dimethylaminobenzaldehyde (20%, w/v; Sigma) for 20 min at 60°C for color development. The optical densities were measured at 557 nm with SaFire (TECAN) microplate reader. HYP content was estimated by linear interpolation using trans-4-hydroxy-L-proline (Sigma) as standard.

### Type II collagen ELISA

Type II collagen ELISA (Chondrex) was used to determine the amount of human type II collagen in microspheres produced by hMSCs according to manufacturer’s instruction. Samples were digested by pepsin in 0.5 N acetic acid with ratio of collagen:pepsin = 10:1 (w/w) for 7 days at 4°C. Supernatant was separated from insoluble residues by centrifugation at 15000 rpm for 15 min. Then samples and standards were placed in 96-well plate pre-coated with capture antibodies (details in manufacturer’s instruction) and detection antibody was added. The plate was incubated for 2 h at room temperature and then washed and incubated with streptavidin peroxidase for 1 h. The plate was washed and OPD solution was added for 30 min at room temperature. Reaction was stopped by 2 N sulfuric acid and the amount of type II collagen was measured by absorbance at 490 nm. Samples were running in quadruplicates.

### RNA isolation and quantitative real time-PCR

Total RNA was extracted using TRI reagent (Molecular Research Center, Inc., Cincinnati, OH) according to the manufacturer’s instructions. RNA was quantified using a NanoDrop-2000 spectrophotometer (NanoDrop, Rockland, DE) and was transcribed into cDNA using a TaqMan Reverse Transcription kit (Applied Biosystems, Foster City, CA). Gene expression levels of collagen type I (COL1A2), aggrecan (ACAN), SRY (sex-determining region Y)-box 9 (SOX9), matrix metalloproteinase-13 (MMP-13), matrix metalloproteinase-2 (MMP-2) and housekeeping gene eukaryotic 18s rRNA were determined by an ABI StepOne Plus real-time PCR system using TaqMan^®^ Gene Expression Master Mix (Applied Biosystems) using standard thermal conditions. Assay IDs and Reference Sequence database accession numbers are listed in Table below. Power SYBR^®^ Green PCR Master Mix (Applied Biosystems) was used for detection of collagen type II (COL2A1), collagen type X (COL10A1) using glyceraldehydes-3-phosphate dehydrogenase (GAPDH) as the housekeeping gene. The sequences of primers used in real time PCR were listed in Tables 3 and 4.

**Table 3:** The primer sequences of genes tested in real time-PCR analysis.

Gene Symbol	Protein Product	Assay ID / Primer sequence
**ACAN**	Aggrecan	Hs00153936_m1
**SOX9**	SRY (sex-determining region Y)-box 9	Hs00165814_m1
**MMP13**	Matrix Metalloproteinase-13	Hs00233992_m1
**Col1A2**	Type I collagen	Hs00164099_m1
**MMP2**	Matrix Metalloproteinase-2	Hs01548727_m1
**18S rRNA**	Ribosomal 18s rRNA	X03205.1 (Accession Number)

**Table 4:** The primer sequences for SYBR green real time-PCR assays.

Gene symbol	Protein Product	Primer sequence
**Col2A1**	Type II collagen	Fw: 5’- TCACGTACACTGCCCTGAAG-3’
		Rv: 5’- TGCAACGGATTGTGTTGTT-3’
**Col10A1**	Type X collagen	Fw:5’-CAGATTTGAGCTATCAGACCAACAA-3’
		Rv:5’-AAATTCAAGAGAGGCTTCACATACG-3’
**GAPDH**	Glyceraldehyde 3-phosphate dehydrogenase	Fw: 5’- GAGTCAACGGATTTGGTCGT-3’
		Rv: 5’-TTGATTTTGGAGGGATCTCG-3’

### Data presentation and statistics

Quantitative data on diameter of the microspheres, collagen content, GAG content, DNA content and expression level of various genes were presented as mean ± SEM. The normality assumption was verified with the Kolmogorov-Smirnov test and the equal variance assumption was verified by Levene’s test to justify the use of parametric tests. Data among different treatment groups at different time points was compared using two-way ANOVA with appropriate post-hoc tests. For data with equal variance assumed, Bonferroni’s test was used. For data without equal variances, Dunnett’s T3 test was used. SPSS 19.0 was used to execute all analyses and the statistical significance was set at α = 0.05.

## Results

### Collagen remodeling by encapsulated hMSCs with and without chondrogenesis

**Fig 1:**
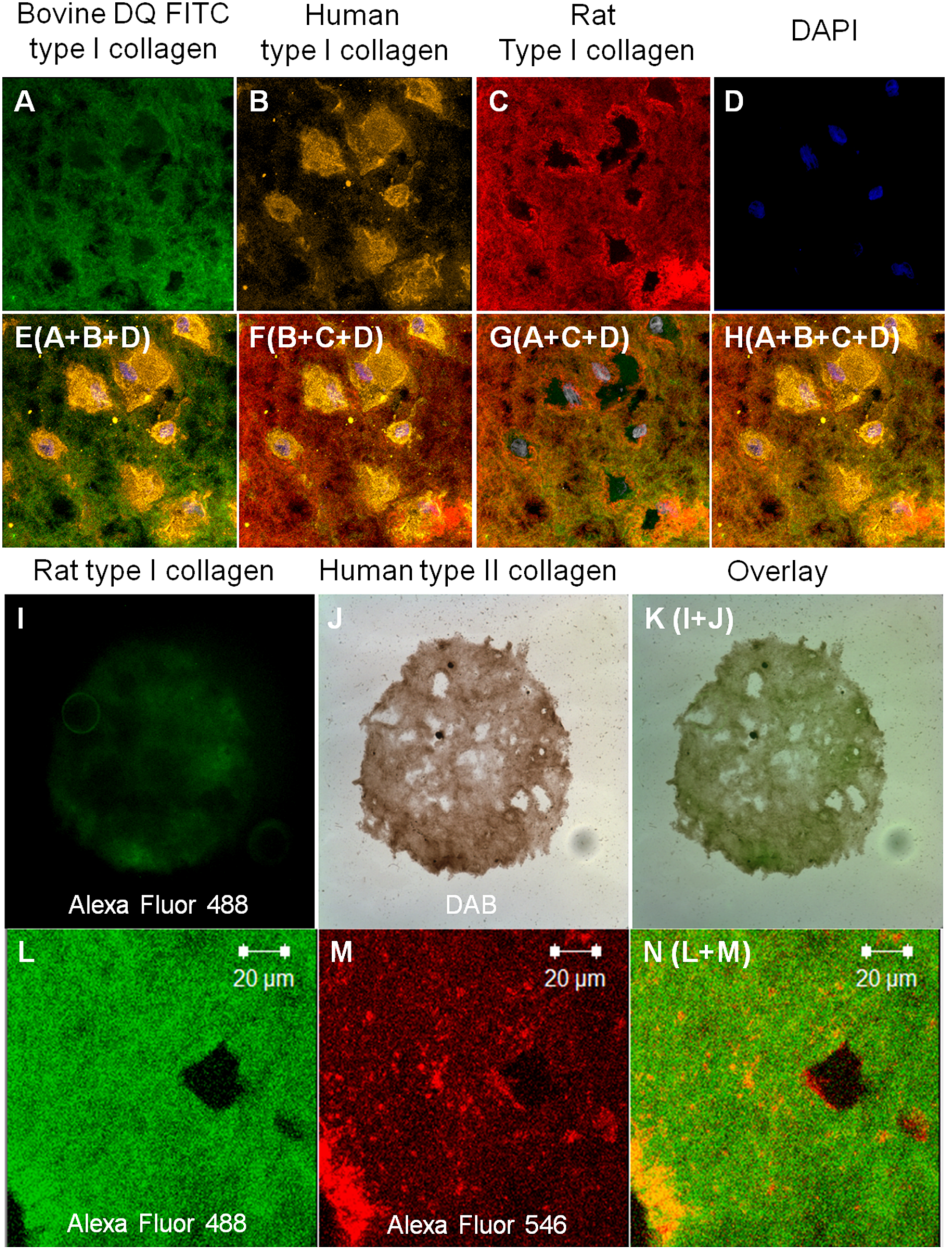
Collagen remodeling in hMSC in rat collagen microspheres. (A-H) Collagen type I remodeling by microencapsulated-hMSCs after 24 hours of incubation. Green: Bovine DQ FITC collagen type I 10 mg/ml, Orange: Human type I collagen, Red: Rat type I collagen 1 mg/ml and Blue: DAPI. (A) Fluorescent staining of bovine DQ FITC type I collagen, which was mixed with rat type I collagen during fabrication of microspheres and it fluoresces if degraded; (B) Immunofluorescent staining of human type I collagen, which should be synthesized by hMSC; (C) Immunofluorescent staining of rat type I collagen; (D) DAPI, which labelled the nuclei; (E) Merged panels A+B+D (cell nuclei in degrading DQ collagen and newly synthesized human type I collage); (F) Merged panels B+C+D (cell nuclei in starting material rat collagen and newly synthesized human type I collagen); (G) Merged panels A+C+D (starting materials rat type I collagen and DQ collagen are largely co-localizing); (H) Merged panels A+B+C+D (cells synthesizing human type I collagen in starting materials, which are undergoing degradation); (I-N) Collagen type II deposition in MSC-collagen type I microsphere after chondrogenic differentiation for 21 days; (I) Immunofluorescent staining of rat type I collagen, which is the starting material of the microsphere; (J) Immunohistochemistry of human type II collagen (DAB: substrated), which is newly synthesized by MSC during chondrogenic differentiation; (K) Merged panels (I+J); (L) Alexa fluor 488 labelled rat type I collagen; (M) Immunofluorescent staining of human type II collagen; (N) Merged panels (L+M) showing co-localization in some regions.

Remodeling refers to simultaneous degradation and synthesis of extracellular matrices (Fig 1A–1H). In collagen encapsulated hMSCs without chondrogenesis induction, remodeling happens as soon as cell encapsulation starts, that is when cells start to interact with collagen gel, degradation starts. Degradation was demonstrated by presence of fluorescence signals of DQ collagen, which turns fluorescent when collagen is being degraded (Fig 1A). The degraded collagen (green channel) was largely co-localizing with (Fig 1G) the fluorescence staining of the rat type I collagen (Fig 1C), which refers to the starting material. The extracellular matrix is being remodeled by hMSCs as simultaneous synthesis of human type I collagen was shown by the intense immunofluorescence staining of human type I collagen at the intracellular space (Fig 1B) and the slight extracellular deposition overlaying with both DQ collagen (Fig 1E) and rat collagen (Fig 1F). During chondrogenesis of hMSC in the 3D collagen microspheres, remodeling of the matrix was also demonstrated (Fig 1I–1N). Firstly, newly synthesized and deposited human type II collagen was found (Fig 1J), it largely co-localized with (Fig 1K) the immunofluorescent staining of rat type I collagen (Fig 1I), the starting material. Secondly, Alexa488 fluorescence labeled rat type I collagen (Fig 1L) largely co-localized (Fig 1N) with immunofluorescent staining of human type II collagen (Fig 1M) at certain regions particularly pericellular and extracellular space, suggesting that chondrogenically differentiating hMSCs used the rat collagen meshwork as the template or scaffold for deposition of new cartilage matrices.

### Inhibiting intracellular and extracellular protease degradation differentially affected chondrogenesis

**Fig 2:**
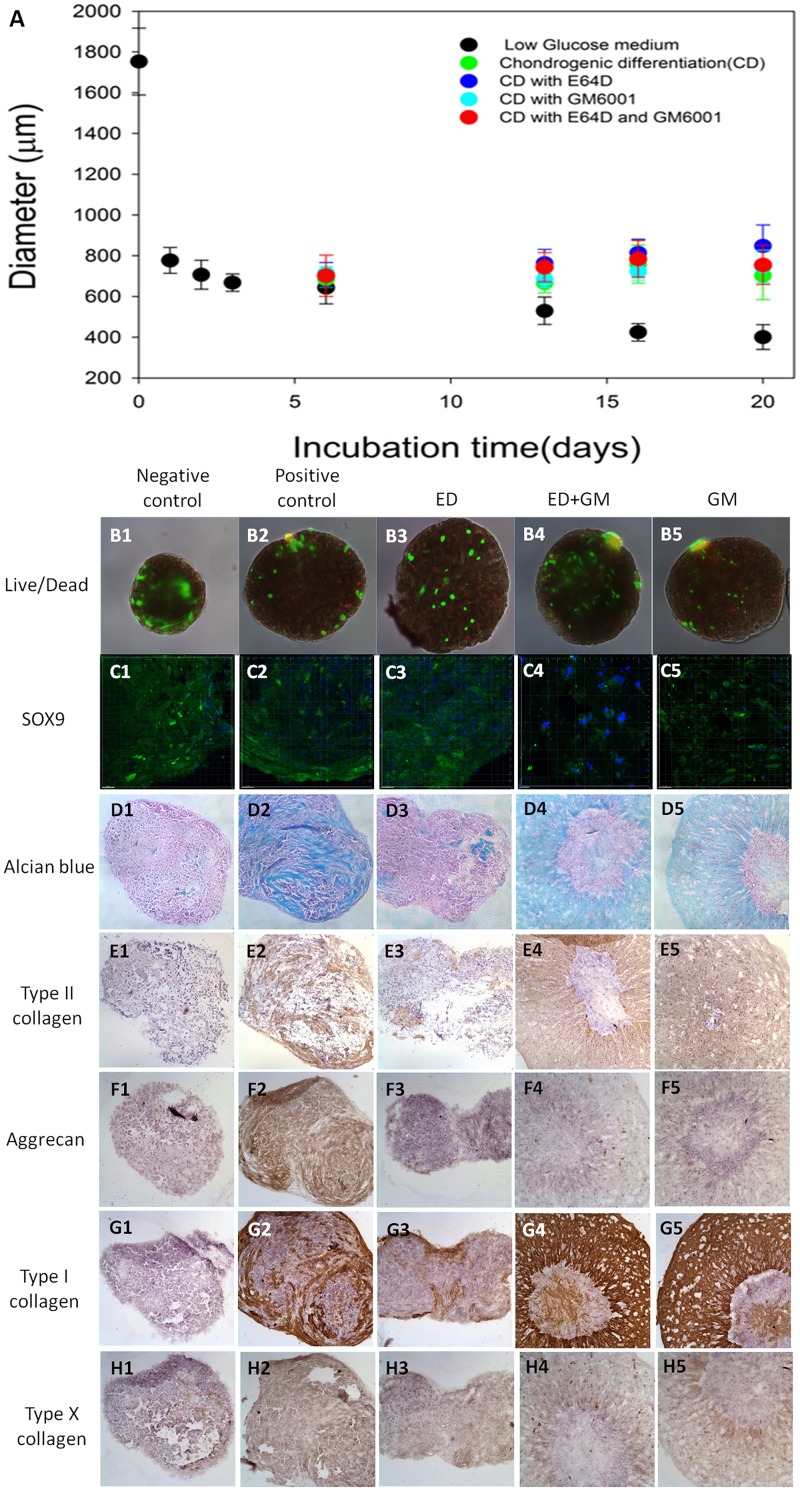
Human MSC-collagen microspheres under cultures. (A) Temporal change in the size of hMSC-collagen microspheres (n = 10); (B1-5) Live and Dead staining of hMSCs in collagen microspheres at day 21 during chondrogenic differentiation (Green: Live cells labeled by cacein AM; Red: Dead cells labeled ethidium homodimer-1); (C1-5) sox 9 immunofluorescence; (D1-5) Alcian blue staining; (E1-5) Type II collagen immunohistochemistry; (F1-5) Aggrecan immunohistochemistry; (G1-5) Type I collagen immunohistochemistry; (H1-5) Type X collagen immunohistochemistry; 1: Negative control (Normal medium); 2: Positive control (Chondrogenic medium with TGFβ alone); 3: ED (Chondrogenic medium with TGFβ and intracellular protease inhibitor E64D); 4: GM (Chondrogenic medium with TGFβ and extracellular matrix protease inhibitor GM6001); 5: ED+GM (Chondrogenic medium with TGFβ and both intracellular protease inhibitor E64D and extracellular protease inhibitor GM6001).

Interfering with protease degradation, both intracellular and extracellular ones, reduced the extent of encapsulation-based contraction of collagen by hMSCs (Fig 2A). Live/Dead staining showed that the cell viability of hMSCs after chondrogenesis did not significantly vary among different groups (Fig 2B1–2B5). Sox9 is an early chondrogenic marker and has been found expressing in all groups (Fig 2C1–2C5) including the normal medium negative control, suggesting that the collagen microencapsulation process alone (Fig 2C1) may induce hMSC chondrogenesis. Alcian blue staining showed the extracellular matrix glycosaminoglycan (GAG) in the hMSCs-encapsulated microspheres (Fig 2D1–2D5) where intensive staining was found in the chondrogenesis group with TGFbeta (positive control) (Fig 2D2) and the chondrogenesis group in the presence of extracellular matrix protease inhibitor groups (Fig 2D4 and 2D5). However, no GAG staining was found in the normal medium negative control group (Fig 2D1) while a few GAG-rich nodules were found in the chondrogenesis group with the intracellular protease inhibitor (Fig 2D3). Similar trend was found in the immunohistochemical staining of the specific extracellular matrix marker of chondrogenesis type II collagen (Fig 2E1–2E5) where positive staining was found in the chondrogenesis positive control group (Fig 2E2), as well as those with the presence of extracellular protease inhibitors (Fig 2E4 and 2E5). No type II collagen was identified in the negative control group (Fig 2E1) while a few nodules with positive type II collagen staining were identified in the intracellular protease inhibitor group (Fig 2E3). Aggrecan was only observed in the positive control group (Fig 2F2) but not other groups (Fig 2F1, 2F3–2F5), suggesting that protease inhibition interfere with the aggrecan deposition. Type I collagen was found in all groups (Fig 2G2–2G5) except negative control group (Fig 2F1) while significantly more type I collagen was deposited in the groups with extracellular protease inhibitors (Fig 2G4 and 2G5) than chondrogenesis group (Fig 2G2). The hypertrophy marker type X collagen was found in all groups but expressed at a minimal intensity (Fig 2H1–2H5).

### Inhibiting protease degradation altered the ECM composition and cellularity

**Fig 3:**
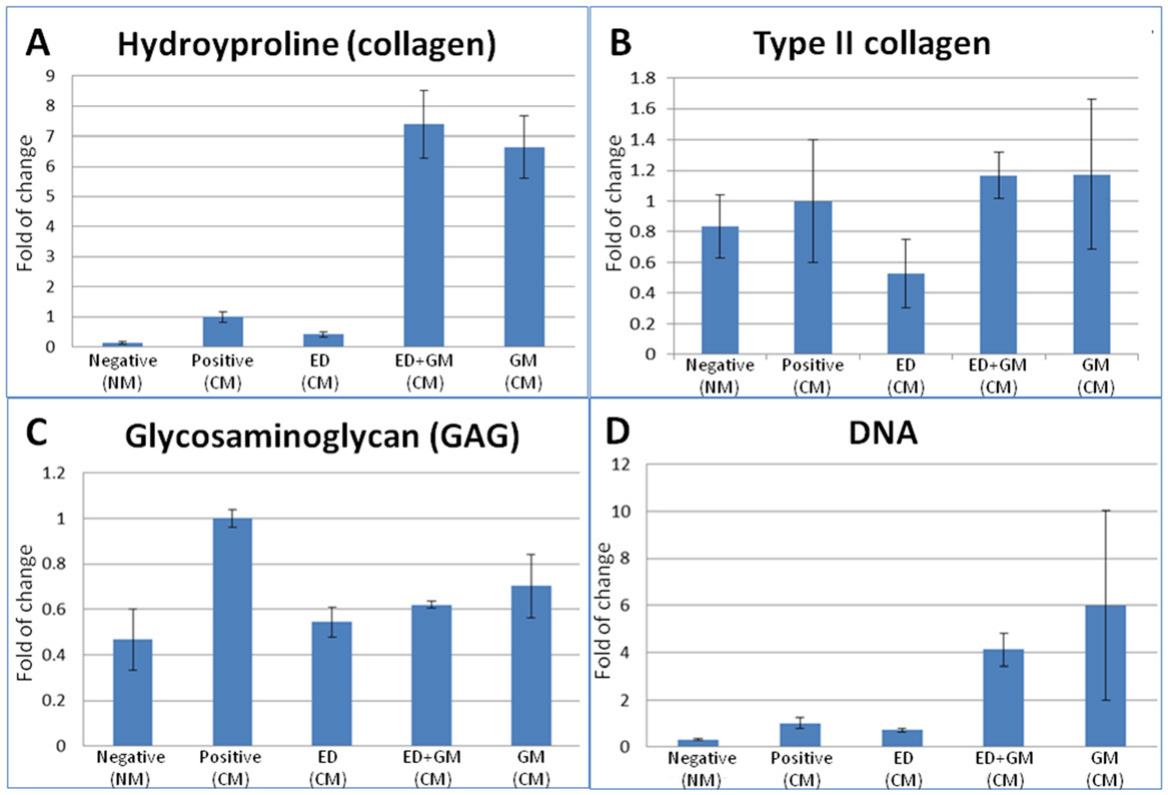
Bar charts showing the extracellular matrix composition of hMSC-collagen microspheres after chondrogenesis. (A) Hydroxyproline (collagen) content (n = 3); (B) Type II collagen content, measured using ELISA (n = 4); (C) Glycosaminoglycan (GAG) content (n = 3); and (D) DNA content (cellularity) (n = 3).

Treating the chondrogenically differentiating hMSC-collagen microspheres with intracellular and extracellular protease inhibitors resulted in very different extracellular matrix compositions (Fig 3) (one-way ANOVA, p<0.001). Fig 3A showed the total collagen content in various groups. When intracellular protease inhibitor ED64 alone was supplemented, there was a 50% reduction in the HYP or total collagen content but the difference was not statistically significant (Dunnet T3 posthoc test, p = 0.088). On the other hand, when extracellular protease inhibitor GM6001 was supplemented, alone or in combination with ED64, there was dramatic increase of almost 7 folds in the total collagen content (Dunnett T3 posthoc test, p = 0.037), suggesting that there was a net collagen deposition. When both intracellular and extracellular protease inhibitors were supplemented, the effects of the GM6001 dominated and the total collagen content also increased for 7 folds (Dunnett T3 posthoc test, p = 0.033). Fig 3B showed the type II collagen content in various groups but one-way ANOVA did not show any statistical significant difference (p = 0.063). The intracellular protease inhibitor reduced the collagen type II content for about 50% while groups with extracellular protease inhibitor, either alone or together with the intracellular protease inhibitor, showed similar type II collagen content as that of the positive control (p≥0.986), corroborating with the immunohistochemistry results in Fig 2E. The differential deposition between total collagen and type II collagen to extracellular protease inhibitor suggested that the 7 fold of increase in total collagen content was not attributable to the Type II collagen, but likely to be type I collagen, as indicated by the immunohistochemistry results in Fig 2G. Fig 3C showed the GAG content. All groups including the intracellular and extracellular protease inhibitors have shown some reduction in GAG content comparing with that of the positive control but the difference was not statistically significant (one way ANOVA, p = 0.951). Fig 3D showed the DNA content. Oneway ANOVA showed no significant difference among different groups (p = 0.091). Groups with the presence of extracellular protease inhibitor showed some increase but the difference was not statistically significant (p≥0.203).

### Inhibiting protease degradation altered the gene expression of chondrogenic markers of hMSCs during chondrogenic induction in collagen microspheres

**Fig 4:**
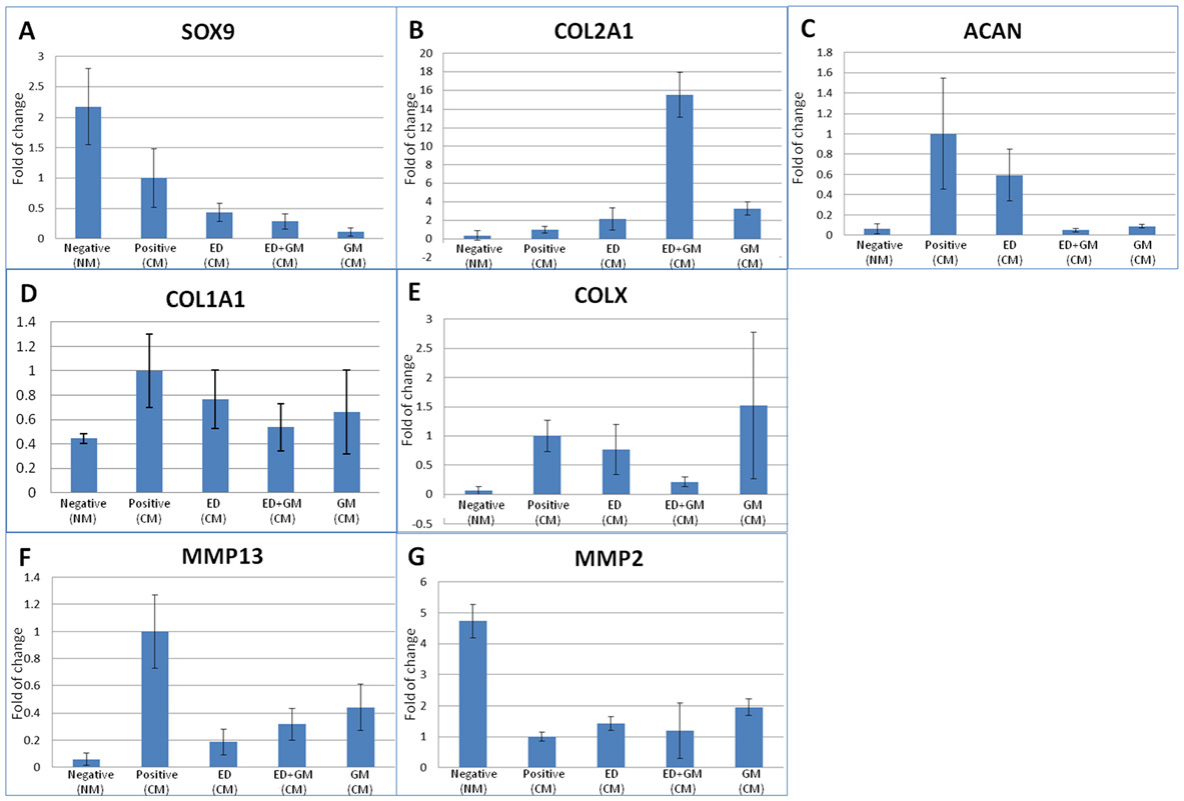
Bar charts showing the expression level of genes related to chondrogenesis. (A) SOX9 (gene of sox9); (B) COL2A1 (gene of type II collagen); (C) ACAN (gene of aggrecan); (D) COL1A1 (gene of type I collagen); (E) COLX (gene of type X collagen); (F) MMP13 (gene of matrix metalloprotease 13); (G) MMP2 (matrix metalloprotease 2 or gelatinase A) (n = 3 in triplicates).

SOX9 is the master chondrogenesis transcription factor (Fig 4A). Oneway ANOVA showed significant difference among various groups (p<0.001) but Dunnett T3 posthoc tests did not reveal this difference as statistically significant (p≥0.104). COL2A1 is the most specific extracellular matrix gene expression marker for chondrogenesis. There was significant difference among different groups (Fig 4B) (Oneway ANOVA, p<0.001). Specifically, there was a dramatic increase of >15 fold in the COL2A1 gene expression when both ED and GM were present (Bonferroni’s posthoc test, p<0.001), suggesting synergistic interactions of intracellular and extracellular protease inhibitors. Nevertheless, the upregulated gene expression of COL2A1 was not reflected in the immunohistochemistry staining in Fig 2E4 and ELISA in Fig 3B. Moreover, although ED alone increased for 2 folds and GM alone for 3.5 folds, the differences were not statistically significant (p≥0.53). Aggrecan is one major proteoglycans synthesized during chondrogenesis although undifferentiating hMSCs have been shown to synthesize aggrecan as well [14]. Oneway ANOVA showed significant difference in ACAN gene expression among different groups (Fig 4C) (Oneway ANOVA, p = 0.005). Comparing with the chondrogenesis positive control, the aggrecan gene (ACAN) was significantly reduced in the negative control (Dunnett T3 posthoc tests, p = 0.017), and the groups with the presence of extracellular protease inhibitors (Dunnett T3 posthoc tests, p≤0.020). Type I collagen (COL1A1) showed slightly reduced expression in groups treated with protease inhibitors (Fig 4D) but the difference was not statistically significant (Oneway ANOVA, p>0.05). Type X collagen (COLX) and MMP13 are regarded as the hypertrophic markers, which are usually upregulated when chondrocytes undergo terminal differentiation to hypertrophic chondrocytes. There was no significant change in the COLX expression in groups with either intracellular or extracellular protease inhibitor (Fig 4E) (Oneway ANOVA, p = 0.087). MMP13 is a matrix protease selectively digesting type II collagen and its expression is related to hypertrophy [15] and cartilage degeneration [16]. Comparing with chondrogenesis positive control, all groups including the protease inhibitor groups and the negative control all significantly down regulated MMP13 expression (Fig 4F) (Oneway ANOVA, p<0.001, Bonferroni’s posthoc tests, p≤0.016). Taking together the results of COLX and MMP13 expression, treating hMSCs during chondrongenesis with protease inhibitors did not promote hypertrophy. MMP2 is gelatinase which digests collagen after activation by the action of membrane bound MMPs such as MMP14 [17]. There was no significant difference in MMP2 expression in all groups (Fig 4G) (Oneway ANOVA, p = 0.352).

## Discussion

The current study demonstrates that inhibiting matrix proteases affects the outcomes of chondrogenic differentiation of human MSCs primarily through an enhanced collagen matrix deposition. In our previous study, the collagen microencapsulation platform has been shown to be chondroconductive that hMSCs entrapped in collagen microspheres were able to differentiate towards the chondrogenic lineages by expressing chondrogenic markers and remodeling the template type I collagen matrix meshwork with a cartilage-specific matrix rich in type II collagen and aggrecan [9]. In the current study, we further demonstrate that hMSCs utilized the rat tail type I collagen meshwork in the microsphere as the template for deposition of the new human type II collagen matrix as shown by their co-localization. Extracellular matrix remodeling is important in tissue dynamic states including development, homeostasis and regeneration, it is therefore logical to hypothesize that manipulating the matrix remodeling process through the inhibition of matrix proteases will affect the outcomes of chondrogenic differentiation of hMSCs in this 3D platform.

Table 5 summarizes the overall effects of intracellular and extracellular protease inhibitors on hMSC chondrogenesis. Inhibiting protease degradation in general reduces the gene expression of the major chondrogenic transcription factor SOX9 and this is particularly effective when extracellular protease inhibitor was used although the sox 9 staining did not alter much. Inhibiting the extracellular proteases showed a significant increase in the total collagen content and the type I collagen staining although its gene expression did not alter much. On the other hand, type II collagen gene expression is dramatically upregulated even though its protein expression did not change significantly. Our in house data in separate study demonstrate that exposing hMSC pellet culture to protease inhibitors in time result in enhanced expression of type I and type II collagen, and aggrecan although GAG content was decreased (S1 Fig). These changes suggest that hMSCs commit towards a fibrocartilage phenotype under the influence of matrix protease inhibition during chondrognesis. Bertram et al. [18] found that broad spectrum pan-MMP inhibitors suppressed all chondrogenesis markers including SOX9, type II collagen and proteoglycans while our study confirmed the reduction in sox9 and aggrecan gene expression but showed enhanced type II collagen gene expression. This discrepancy may be due to the difference in the *in vitro* chondrogenic models used. A spheroid model was used in that study while the current model uses a collagen microsphere model. This suggests that the presence of extracellular matrix in the 3D model might interfere with the responses of hMSC to protease inhibitors. Previous studies have demonstrated that integrin binding is involved in MSC chondrogenesis. For example, hMSCs encapsulated in hydrogel incorporated with collagen mimetic peptide capable of binding cells via β1 integrins showed greater extent of chondrogenic differentiation than in hydrogel alone [19]. Blockage of β1 integrin reduced gene expression of chondrogenic markers by hMSCs encapsulated in hydrogel with type I collagen incorporation [20]. Mhanna et al. [21] also demonstrated that hydrogel modified with collagen mimetic peptide favored GAG production and gene expression of type II collagen and aggrecan, compared to hydrogel modified with fibronectin adhesion peptide. The current collagen microencapsulation platform imposed high density collagen ligands and hence subsequent binding and signaling to hMSCs during chondrogenesis that may help to promote collagen deposition. A high type I collagen deposition is regarded as a typical feature in fibrocartilage [22]. Fibrocartilage tissues such as annulus fibrosus and menisci are typically characterized by high levels of type I collagen as well as the presence of proteoglycans [23]. A large variety of biochemical and mechanical stimuli have been applied in fibrocartilage tissue engineering [24]. The potential of manipulating protease inhibitors to promote fibrocartilage formation for intervertebral disc and meniscus tissue engineering needs to be further investigated.

**Table 5:** Summary on the gene and protein expression of chondrogenic markers in chondrogenically differentiating hMSCs in collagen microspheres under different protease inhibitor treatment.

Chondrogenic markers		Treatment groups			
		Positive	ED	ED+GM	GM
**Sox 9**	Sox 9 staining	—	—	—	—
	SOX9 gene	—	↓	↓	↓↓
**Total collagen**	HYP content	—	—	↑↑	↑↑
**Col I**	Col I staining	—	↓	↑	↑
	Col I gene	—	—	—	—
**Col II**	Col II staining	—	↓	—	—
	Col II ELISA	—	—	—	—
	Col II gene	—	↑	↑↑	↑
**Proteoglycan**	GAG staining	—	↓	—	—
	GAG content	—	—	—	—
	Aggrecan staining	—	↓	—	—
	ACAN gene	—	—	↓	↓
**Col X**	Col X gene	—	—	—	—
**MMP13**	MMP13 gene	—	↓	↓	↓
**MMP2**	MMP2 gene	—	—	—	—

“—”denotes similar expression; “↓” denotes downregulation; “↓↓” denotes extensive downregulation; “↑” denotes upregulation; “↑↑” denotes intensive upregulation.

It is interesting to note that manipulating collagen remodeling using protease inhibitors also partially affects the proteglycan metabolism. Specifically, gene expression of the major cartilage proteoglycan aggrecan was downregulated, although the protein expression and content was not significantly changed. This suggests possible interactions between the collagen deposition and proteoglycan regulation. It is generally known that collagen fibrillogenesis can be regulated by proteoglycans particularly the small leucine-rich proteoglycans (SLRPs) including decorin, aspirin and fibromodulin [25, 26]. Moreover, interactions of matrix metalloproteinases (MMPs) for collagen and aggrecanases (e.g. ADMATSs) for aggrecans in regulating extracellular matrix remodeling have been suggested in musculoskeletal tissue pathology such as intervertebral disc degeneration [27]. The crosstalk between collagen deposition upon protease inhibition and proteoglycan expression warrants further investigation before targeting these enzymes for promoting extracellular matrix deposition during tissue engineering.

## Conclusions

The current study demonstrated that inhibition of extracellular proteases affects chondrogenic differentiation of MSCs by favoring the phenotype of fibrocartilage with upregulated type I collagen deposition and type II collagen gene expression, without obvious change in the gene expression of the hypertrophy marker type X collagen. This study suggests the potential of manipulating extracellular proteases to differentiate hMSCs towards fibrocartilage formation in intervertebral disc and meniscus tissue engineering.

## Supporting Information

S1 Fig Human MSC pellet culture. A-E: Alcian blue staining of hMSC pellet culture at day 21; F-J: Dual channel immunofluorescent staining of type I collagen (green) and sox 9 (red); K-O: Dual channel immunofluorescent staining of type II collagen (green) and aggrecan (red); A, F, K: Normal medium; B, G, L: Chondrogenic medium; C, H, M: Chondrogenic medium with intracellular protease inhibitor E64D; D, I, N: Chondrogenic medium with extracellular matrix protease inhibitor GM6001; E, J, O: Chondrogenic medium with both E64D and GM6001.
(TIF)
