# Supplementary figures and images for: Extracellular Protease Inhibition Alters the Phenotype of Chondrogenically Differentiating Human Mesenchymal Stem Cells (MSCs) in 3D Collagen Microspheres
Source: PLoS One. 2016 Jan 13;11(1):e0146928. doi: 10.1371/journal.pone.0146928 (PMC4711899; doi:10.1371/journal.pone.0146928)

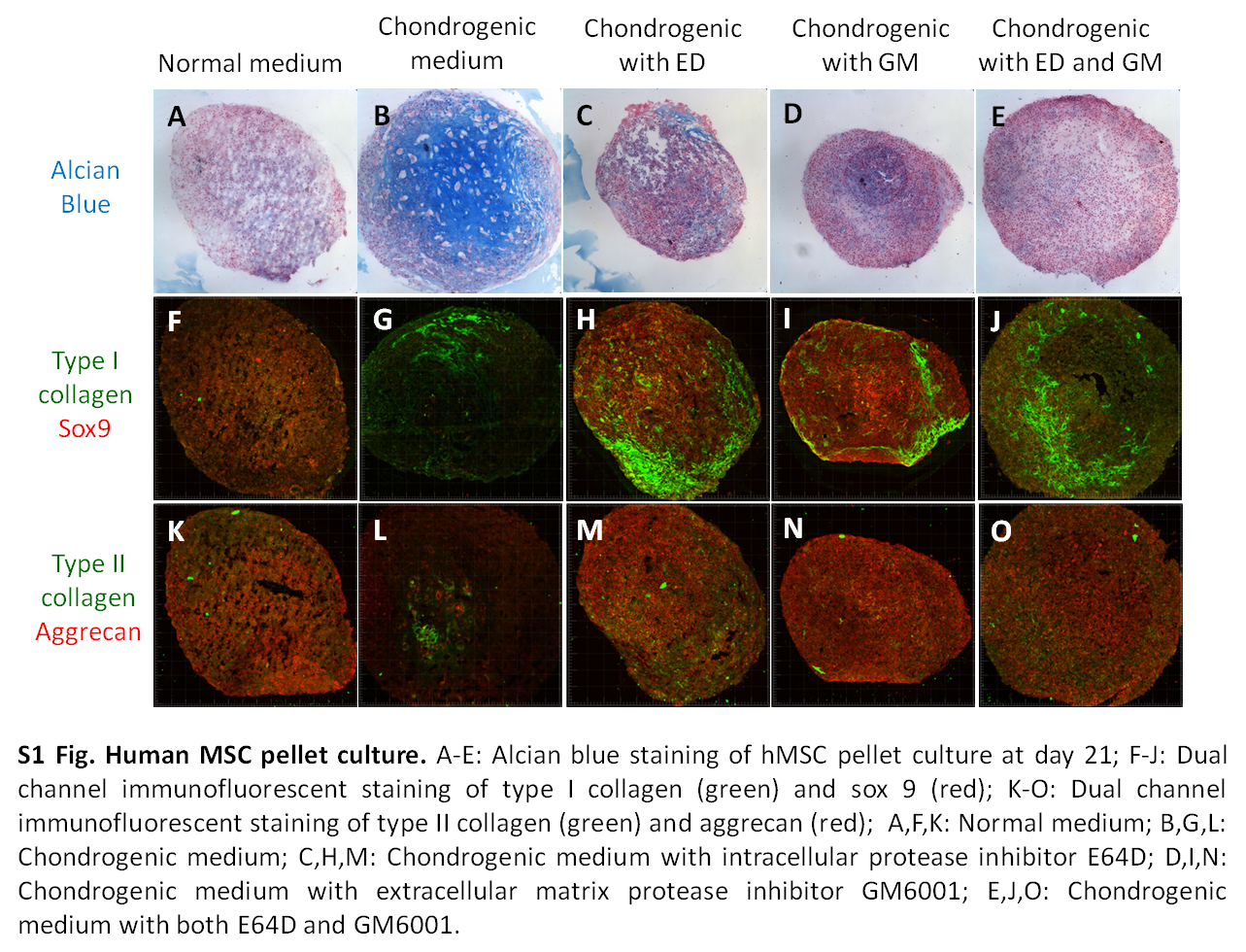

Supplement: S1 Fig — A-E: Alcian blue staining of hMSC pellet culture at day 21; F-J: Dual channel immunofluorescent staining of type I collagen (green) and sox 9 (red); K-O: Dual channel immunofluorescent staining of type II collagen (green) and aggrecan (red); A, F, K: Normal medium; B, G, L: Chondrogenic medium; C, H, M: Chondrogenic medium with intracellular protease inhibitor E64D; D, I, N: Chondrogenic medium with extracellular matrix protease inhibitor GM6001; E, J, O: Chondrogenic medium with both E64D and GM6001. (TIF) [file pone.0146928.s001.tif]
